# Supplementary material for: Molecular Cloning and Identification of NADPH Cytochrome P450 Reductase from Panax ginseng
Source: Molecules. 2021 Nov 3;26(21):6654. doi: 10.3390/molecules26216654 (PMC8588036; doi:10.3390/molecules26216654)
Supplement: Supplementary file 1 [file molecules-26-06654-s001.zip › molecules-1383173-supplementary.pdf]

molecules

Article

Molecular cloning and identification of NADPH cytochrome P450 reductase from *Panax ginseng*

Xian Zou, Yue Zhang, Xu Zeng, Tuo Liu , Gui Li, Yuxin Dai, Yuanzhu Xie and Zhiyong Luo*

[1] Department of Biochemistry and Molecular Biology, School of Life Sciences, Central South University, Changsha 410008, China; 172511011@csu.edu.cn (X.Z.); zhang1045242781@126.com(Y.Z.); xuzeng_katharine@163.com (X.Z.); lt1994@csu.edu.cn (T.L.); ligui20061029@126.com (G.L.); yuxin_dai@csu.edu.cn (Y.D.); 172511004@csu.edu.cn (Y.X.)

* Correspondence: luozhiyong@csu.edu.cn; Tel.: +86-731-8480-5449

Supplementary Materials:

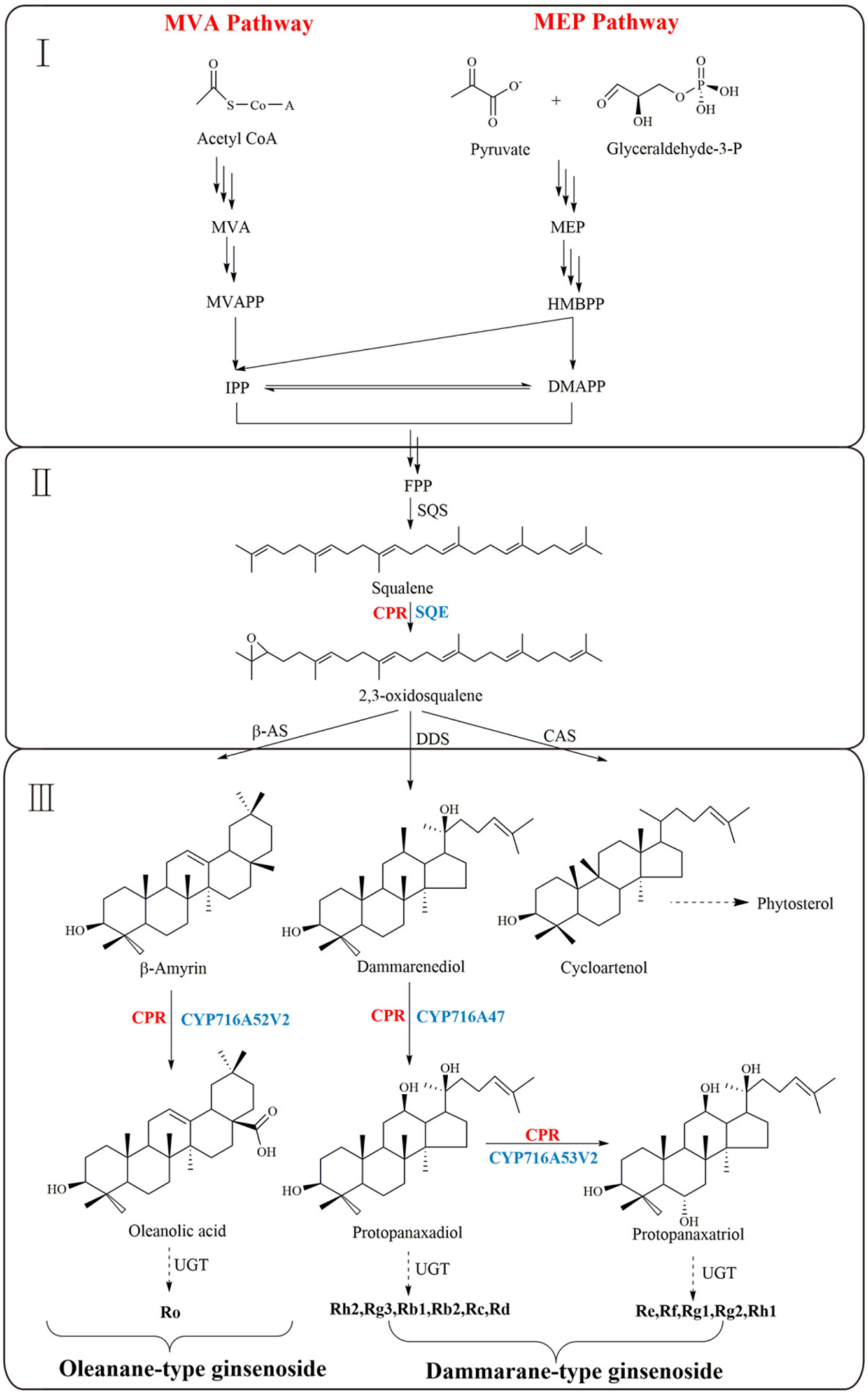

Figure S1. Main characteristic steps of the ginsenoside biosynthesis pathway. Ginsenoside synthesis pathway can consist of three parts. In the first part, is the MVA pathway and the MEP pathway. The second part, the formation of the tetracyclic framework. The third part is the modification of the skeleton. Among them, CYP450s and squalene epoxidase (SQE) play an important role in the ginsenoside synthesis pathway, which is marked in blue. All these enzymes need CPRs as a partner to be presented in red.

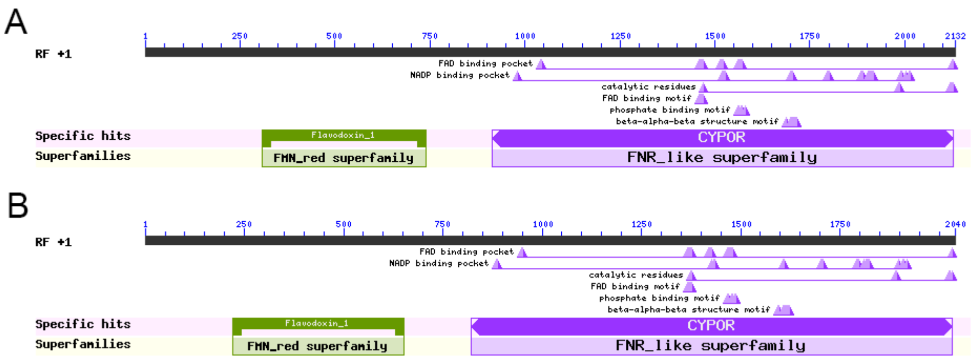

Figure S2. The conserved domains of PgCPR1 (**A**) and PgCPR2 (**B**). Predicted by the NCBI conserved domains finder (https://www.ncbi.nlm.nih.gov/Structure/cdd/wrpsb.cgi).

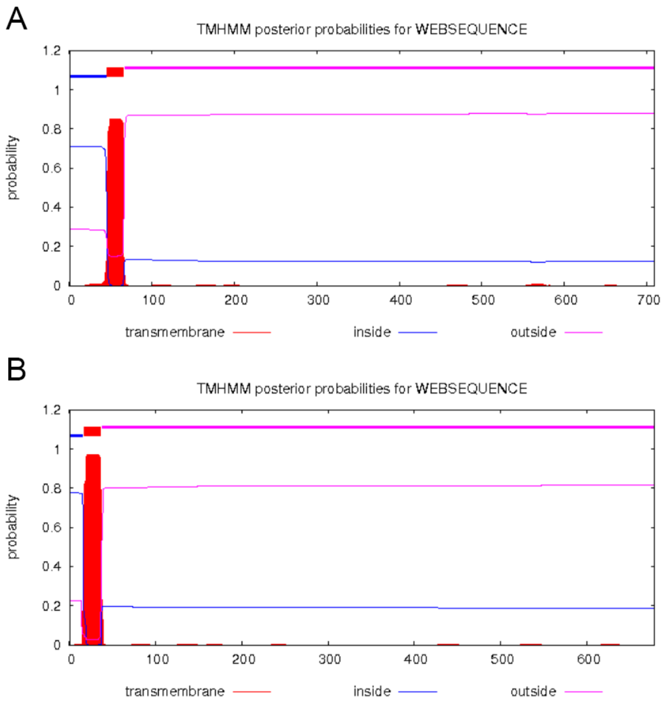

Figure S3. The transmembrane domain of PgCPR1 (**A**) and PgCPR2 (**B**). Predicted by TMHMM Server v. 2.0 (http://www.cbs.dtu.dk/services/TMHMM/).

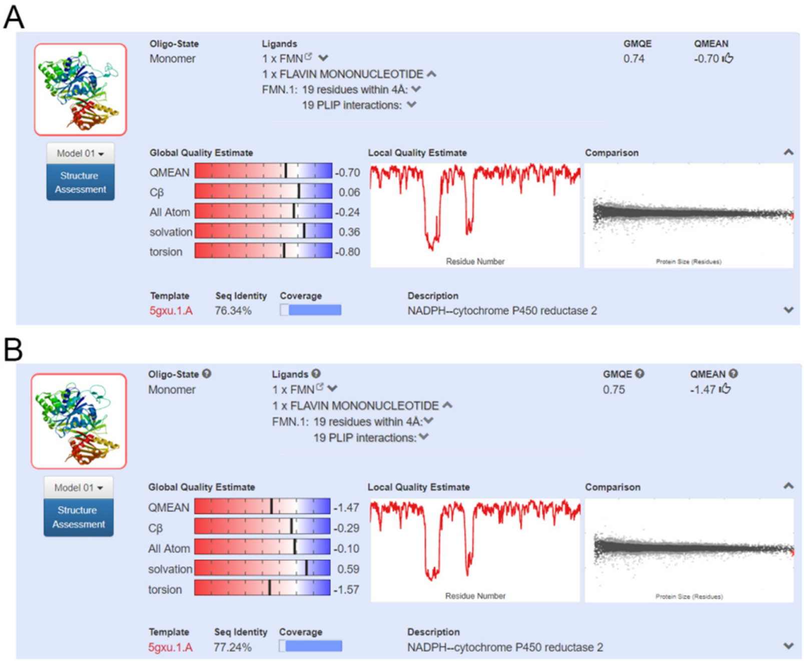

Figure S4. The protein tertiary structure analysis of PgCPR1 (**A**) and PgCPR2 (**B**). Predicted by SWISS-MODEL online tool (https://swissmodel.expasy.org/).

Table S1. List of primers used in this study.

Primers	Sequences(5'-3')	Application
PgCPR1F	ATGCTGAAAGTGTCTCCCTT	CDS cloning
PgCPR1R	TTACCATACATCACGCAGAT	CDS cloning
PgCPR2F	ATGGCTGCGATGCCGACGTC	CDS cloning
PgCPR2R	TTACCATACATCACGCAGAT	CDS cloning
28a-PgCPR1-HindⅢF	cgagtgcggccgcaagcttgCCATACATCACGCAGATATC	Prokaryotic expression
28a-PgCPR1-EcorⅠR	tgggtcgcggatccgaattcCGGAAATCGTCGAGCCAGAA	Prokaryotic expression
28a-PgCPR2-HindⅢF	cgagtgcggccgcaagcttgCCATACATCACGCAGATATC	Prokaryotic expression
28a-PgCPR2-EcorⅠR	tgggtcgcggatccgaattcCGGAGATCGTCGAGCCAGCG	Prokaryotic expression
28a-AtCPR1-HindⅢF	cgagtgcggccgcaagcttgCCAGACATCTCTGAGGTATC	Prokaryotic expression
28a-AtCPR1-EcorⅠR	tgggtcgcggatccgaattcTGGAAGAAAACGACGGCGGA	Prokaryotic expression
PgCPR1-qPCRF	TTTTCGGCACGCAAACTGGTA	Real-Time analysis
PgCPR1-qPCRR	GCTGCATTGTCGGTTGGTTCA	Real-Time analysis
PgCPR2-qPCRF	TGAGAAAACACCTACAGGACGAA	Real-Time analysis
PgCPR2-qPCRR	AAACCCCTAAAAGGAGCCAAT	Real-Time analysis

The lower-case letters were the overlap area of vectors, and the capital letters were the target.

genes.
